# Inhibition of SARS-CoV-2 3CLpro by chemically modified tyrosinase from *Agaricus bisporus*†

**DOI:** 10.1039/d4md00289j

**Published:** 2024-09-16

**Authors:** David Aguilera-Rodriguez, David Ortega-Alarcon, Angela Vazquez-Calvo, Veronica Ricci, Olga Abian, Adrian Velazquez-Campoy, Antonio Alcami, Jose M. Palomo

**Affiliations:** a Instituto de Catálisis y Petroleoquímica (ICP), CSIC C/Marie Curie 2 28049 Madrid Spain josempalomo@icp.csic.es; b Instituto de Investigación Sanitaria Aragón (IIS Aragón) 50009 Zaragoza Spain; c Centro de Investigación Biomédica en Red en el Área Temática de Enfermedades Hepáticas y Digestivas (CIBERehd) 28029 Madrid Spain; d Institute for Biocomputation and Physics of Complex Systems, University of Zaragoza Spain; e Department of Biochemistry and Molecular and Cell Biology, University of Zaragoza Spain; f Centro de Biología Molecular Severo Ochoa, Consejo Superior de Investigaciones Científicas (CSIC)-Universidad Autónoma de Madrid (UAM) 28049 Madrid Spain

## Abstract

Antiviral compounds are crucial to controlling the SARS-CoV-2 pandemic. Approved drugs have been tested for their efficacy against COVID-19, and new pharmaceuticals are being developed as a complementary tool to vaccines. In this work, a cheap and fast purification method for natural tyrosinase from *Agaricus bisporus* (AbTyr) fresh mushrooms was developed to evaluate the potential of this enzyme as a therapeutic protein *via* the inhibition of SARS-CoV-2 3CLpro protease activity *in vitro*. AbTyr showed a mild inhibition of 3CLpro. Thus, different variants of this protein were synthesized through chemical modifications, covalently binding different tailor-made glycans and peptides to the amino terminal groups of the protein. These new tyrosinase conjugates were purified and characterized through circular dichroism and fluorescence spectroscopy analyses, and their stability was evaluated under different conditions. Subsequently, all these tyrosinase conjugates were tested for 3CLpro protease inhibition. From them, the conjugate between tyrosinase and a dextran-aspartic acid (6 kDa) polymer showed the highest inhibition, with an IC_50_ of 2.5 μg ml^−1^ and IC_90_ of 5 μg ml^−1^, with no cytotoxicity activity by polymer insertion. Finally, SARS-CoV-2 virus infection was studied. It was found that this new AbTyr-Dext6000 protein showed an 80% decrease in viral load. These results show the capacity of these tyrosinase bioconjugates as potential therapeutic proteins, opening the possibility of extension and applicability against other different viruses.

## Abstract 2

A novel antiviral and non-cytotoxic bioconjugate of tyrosinase from *Agaricus bisporus* (AbTyr)-dextran-aspartic acid (6 kDa) polymer is developed.

## Graphical abstract

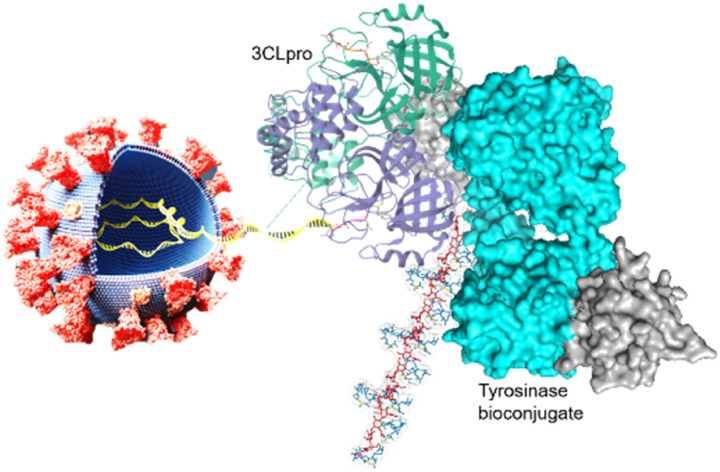

## 1. Introduction

The novel coronavirus SARS-CoV-2 emerged in late 2019 in Wuhan, China, causing a disease that has spread worldwide, termed COVID-19, and was declared a pandemic by the World Health Organization (WHO) in early 2020.^1^ Since then, in parallel with vaccine development, much efforts have been focused on the development of potential antiviral drugs or therapies in treating this disease.^2–6^ Potential therapeutic drugs aim to target proteins involved in essential processes for viral infection and replication. These include cellular host proteins, such as the angiotensin-converting enzyme-related carboxypeptidase (ACE2) receptor or transmembrane protease serine 2 (TMPRSS2) as well as structural viral proteins such as the spike protein.^1^ Non-structural viral proteins are involved in genome and structural protein replication and therefore represent a target for inhibitory drugs.^7^ Among these are RNA-dependent RNA (RNA-DR) proteins. Particularly, RNA-dependent RNA polymerase (RdRp) and 3-chymotrypsin-like protease (3CLpro) have been the focus in drug development because they are essential for viral replication, and there are no similar targets in the host organism.^8^ SARS-CoV-2 contains a large positive single-stranded RNA genome that encodes structural proteins and two overlapping polyproteins (pp1a and pp1ab). 3CLpro is responsible for processing the C-terminal region of these polyproteins, including 11 non-structural proteins.^9,10^

There are two potential approaches to inhibiting 3CLpro: the repurposing of already approved drugs and the use of small molecules or peptides as potential inhibitors, which can be explored *via* high-throughput methods.^7^ Indeed, antiviral drugs that are already approved to treat COVID-19, including remdesivir (Veklury),^11^ nirmatrelvir–ritonavir (Paxlovid),^12^ and molnupiravir (Lagevrio),^13^ are novel peptidomimetics that function as 3CLpro inhibitors.

**Fig. 1:**
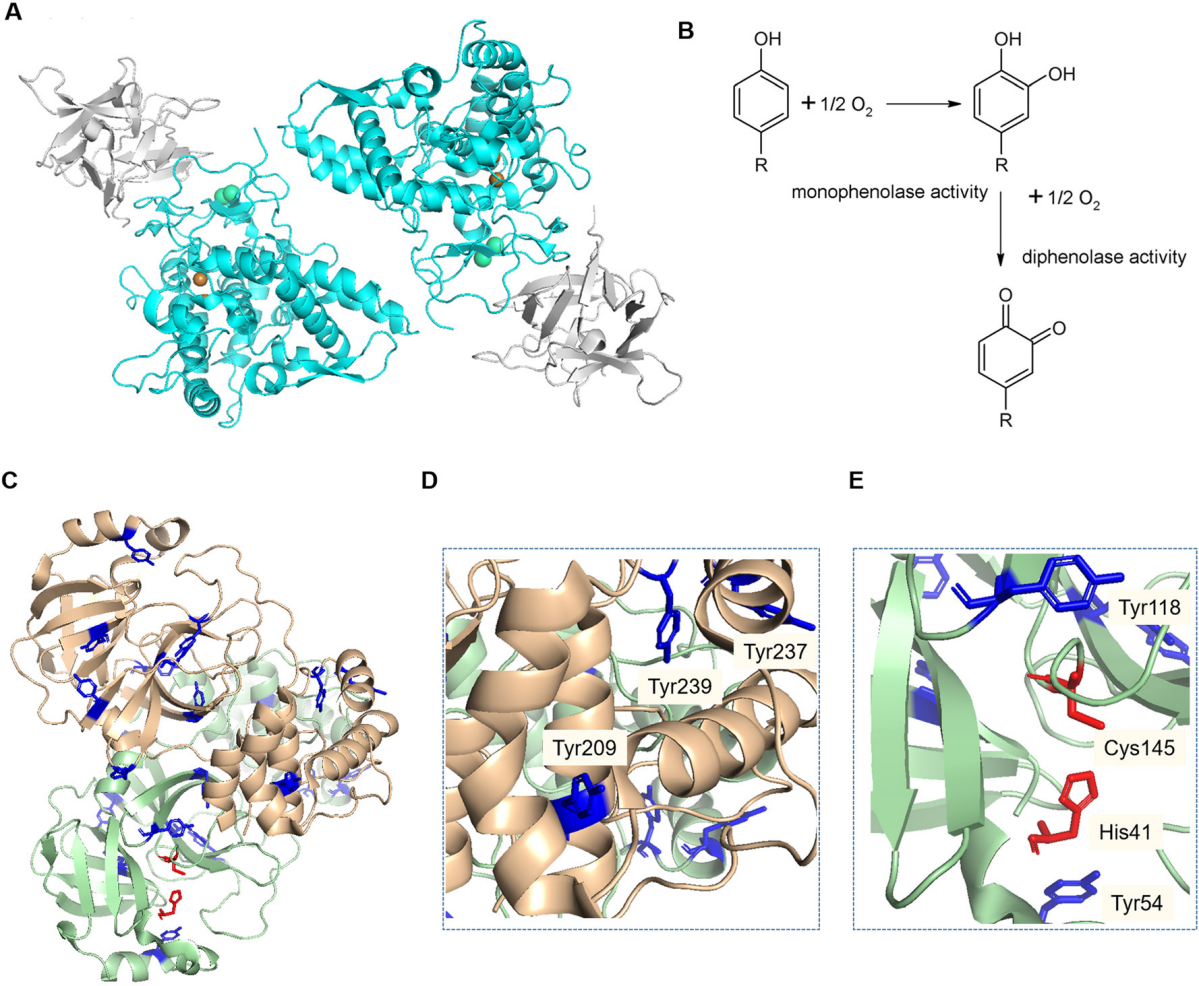
A) Structure of the dimeric form of AbTyr. Protein Data Bank code: 2Y9W; image created using Pymol (H subunits in light blue, L subunits in grey). B). Scheme of reactions catalysed by tyrosinase. C–E). Crystal structure of the SARSCov-2, 3CLpro homodimer. Protein Data Bank code: 7EN8. Image created using Pymol. Tyrosine residues (blue). Catalytic active site residues (red). Tyrosine residues in the area of protein interaction (D). Tyrosine residues near the active site of the 3CLpro structure (E).

Tyrosinase (EC 1.14.18.1) is an enzyme found in an extensive number of species, including humans, animals, plants, bacteria and fungi. In the case of *Agaricus bisporus*, six different genes code for tyrosinase isoforms (*ppo1-6*), of which *ppo3* and *ppo4* have been isolated from fruiting bodies.^14,15^ This enzyme contains two copper atoms in its active site, catalysing the hydroxylation of monophenols and the subsequent oxidation of the diphenols into quinones (Fig. 1B).^16^ Tyrosinase is usually found in a 120 kDa dimeric form, consisting of two subunits H of ∼45 kDa each, and two subunits L of ∼14 kDa. Only the H subunits have been reported to have catalytic activity, and are known to be sufficient for this activity (Fig. 1A).^17^

Recently, we have demonstrated that tyrosinase from *A. bisporus* (AbTyr) shows antiviral activity against the hepatitis C virus (HCV). AbTyr, which exhibits mono and diphenolase activity, (Fig. 1B), was proved to be responsible for the enzymatic antiviral activity, as the inactivated protein did not inhibit viral replication.^18^ This inhibition seems to go through a mechanism based mainly on the hydroxylation of critical tyrosine residues of the HCV protease.

Herein, we hypothesize that AbTyr may inhibit 3CLpro from SARS-CoV-2, a 68.8 kDa homodimer (Fig. 1C), by the hydroxylation of two specific tyrosine residues, Tyr118 and Try54 (Fig. 1D), near the active site (its catalytic dyad consists of His41 and Cys145) of the viral protease, or by disrupting the formation of the homodimer^19^*via* oxidation of three tyrosinases in each monomeric structure (Fig. 1E).

This may be the first example of a therapeutic protein in the treatment of SARS-CoV-2. However, protein-drugs as therapeutic agents have some limitations, such as stability or the efficiency of intracellular delivery to achieve their therapeutic effects. Preparations of bioconjugates *via* covalent protein modification^20,21^ with polymers such as poly(ethylene glycol) (PEG) chains (PEGylation) to reduce immunogenicity and increase the time the therapeutic protein circulates in the bloodstream,^22^ or carbohydrate molecules (dextrans or sialic acids) have been successfully used to improve the protein stability and cell permeability capacity.^23,24^ In the case of enzymes, this protein chemistry also provides bioconjugates with higher enzymatic activity.^25^

Therefore, new bioconjugates of AbTyr, isolated and purified from mushrooms, have been prepared in this work by chemically binding glycans and peptides to the terminal amine groups of this enzyme. These bioconjugates were tested as novel inhibitors of 3CLpro, evaluating the effect of the modification on the antiviral efficiency and cell localization.

## 2. Results and discussion

### 2.1. Protein extraction from mushrooms

**Fig. 2:**
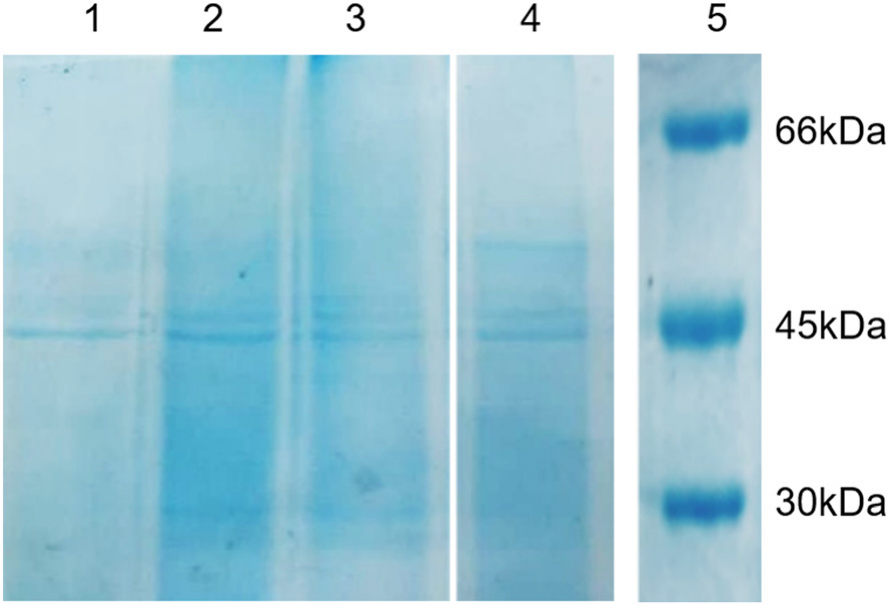
SDS-PAGE gel of mushroom protein extracts. 1: Champinter, 2: Neofungi, 3: Laumont (var. brunnescens), 4: Laumont. 5. Low-molecular weight marker.

To obtain tyrosinase from mushrooms, our previously described protein extraction protocol was performed to first evaluate the efficiency in four mushroom batches from four different commercial sources. The final overall protein production yield in milligrams and the final specific activity of the enzyme against l-DOPA were determined (Table 1). The presence of AbTyr and other proteins in the extracts was validated with SDS-PAGE (Fig. 2).

**Table 1:** Protein yield and specific diphenolase activity obtained from mushrooms

Mushrooms	Protein yield (mg)	Specific activity (U μg^−1^)
Source company		
Champinter	108	1.81
Neofungi	121	3.63
Laumont (var. brunnescens)	182	3.53
Laumont	160	3.23

Most of the sources exhibited a specific activity of around 3.5 U μg^−1^, except the one from Champinter, which showed lower activity (Table 1). After SDS-PAGE electrophoresis of the different protein extracts (Fig. 2), the clear presence of an isoform of AbTyr at 50 kDa was found to be responsible for the superior specific activity values. We have previously demonstrated that this AbTyr isoform has higher activity against l-DOPA than the native AbTyr.^26^

An initial experiment was performed to evaluate the inhibition activity of these protein extracts against the 3CLpro homodimer protease. However, this experiment showed that the samples exhibited intrinsic protease activity, distorting the fluorescence of the FRET assay and highlighting the necessity to further purify the tyrosinase to remove proteases from the mushroom extracts. Mushrooms from Neofungi were selected to perform the following experiments, as their protein extract presented a higher specific activity of AbTyr.

### 2.2. Development of a tyrosinase purification method

**Fig. 3:**
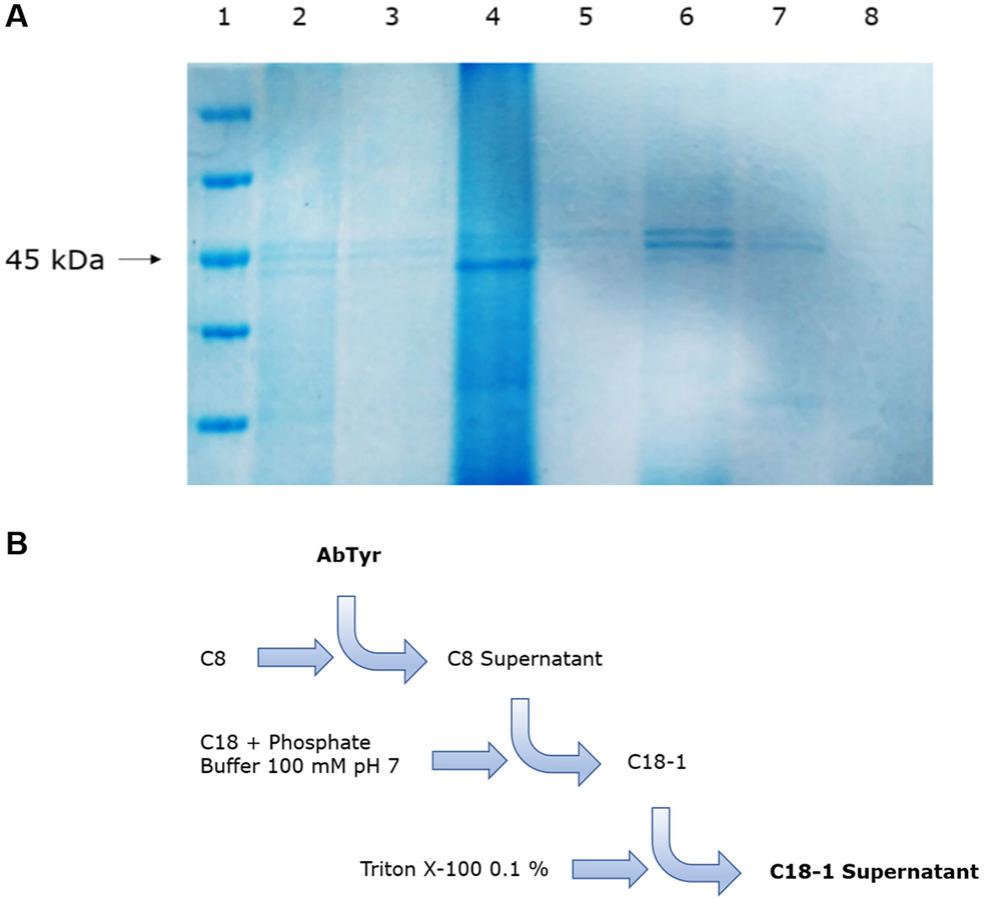
Purification of AbTyr. A. SDS-PAGE of the AbTyr purification cascade. 1: marker, 2: extract, 3: C8 supernatant, 4: C8, 5: C18 supernatant, 6: C18, 7: Triton X-100 elution supernatant, 8: Triton X-100 C18. B. Scheme of the AbTyr purification cascade. C18-1: proteins supported on C18.

Purification of AbTyr from the mushroom extract was firstly performed using a chromatographic hydrophobic adsorption step on different solid supports functionalized with different groups (from octyl to octadecyl groups). The adsorption was tested using the extract directly or by first dialyzing the proteins in sodium phosphate buffer (25 mM), with the purpose of removing ammonium sulfate present from the protein precipitation step of the extraction protocol. In most solid supports, enzyme absorption did not occur in a percentage higher than 40%. However, in the case of C18 in sodium phosphate buffer (0.1 M, pH 7), 80% of the offered protein was bound to the solid phase. Interestingly, when using C8-Sheparose, almost all enzymatic activity remained in the supernatant, while other proteins were retained in the support, as shown in the SDS-PAGE (Fig. 3A).

Thus, a purification cascade approach was finally developed to obtain purified AbTyr. First, 4 mL of dialyzed protein (0.4 mg mL^−1^) in sodium phosphate buffer (25 mM, pH 7) was incubated with Octyl-Sepharose (C8) support for 1 hour, then the supernatant was recovered and incubated with octadecyl-support (C18) in sodium phosphate buffer (100 mM, pH 7) for 1 hour. This step allowed for absorption of the full tyrosinase to the support, separating the other proteins present in the supernatant. Finally, to recover the enzyme in solution, Tyr-C18 derivative was incubated in an aqueous solution of 0.1% (v/v) Triton X-100 (Fig. 3B).

### 2.3. Solid-phase protein modifications using the EDC/NHS strategy

With the objective of enhancing the inhibitory activity of AbTyr against 3CLpro, several modifications were performed using different glycans and peptides. These polymers may help AbTyr to recognize 3CLpro tyrosine residues by allosteric reasons and/or because of charges interacting with nearby amino acids.

**Fig. 4:**
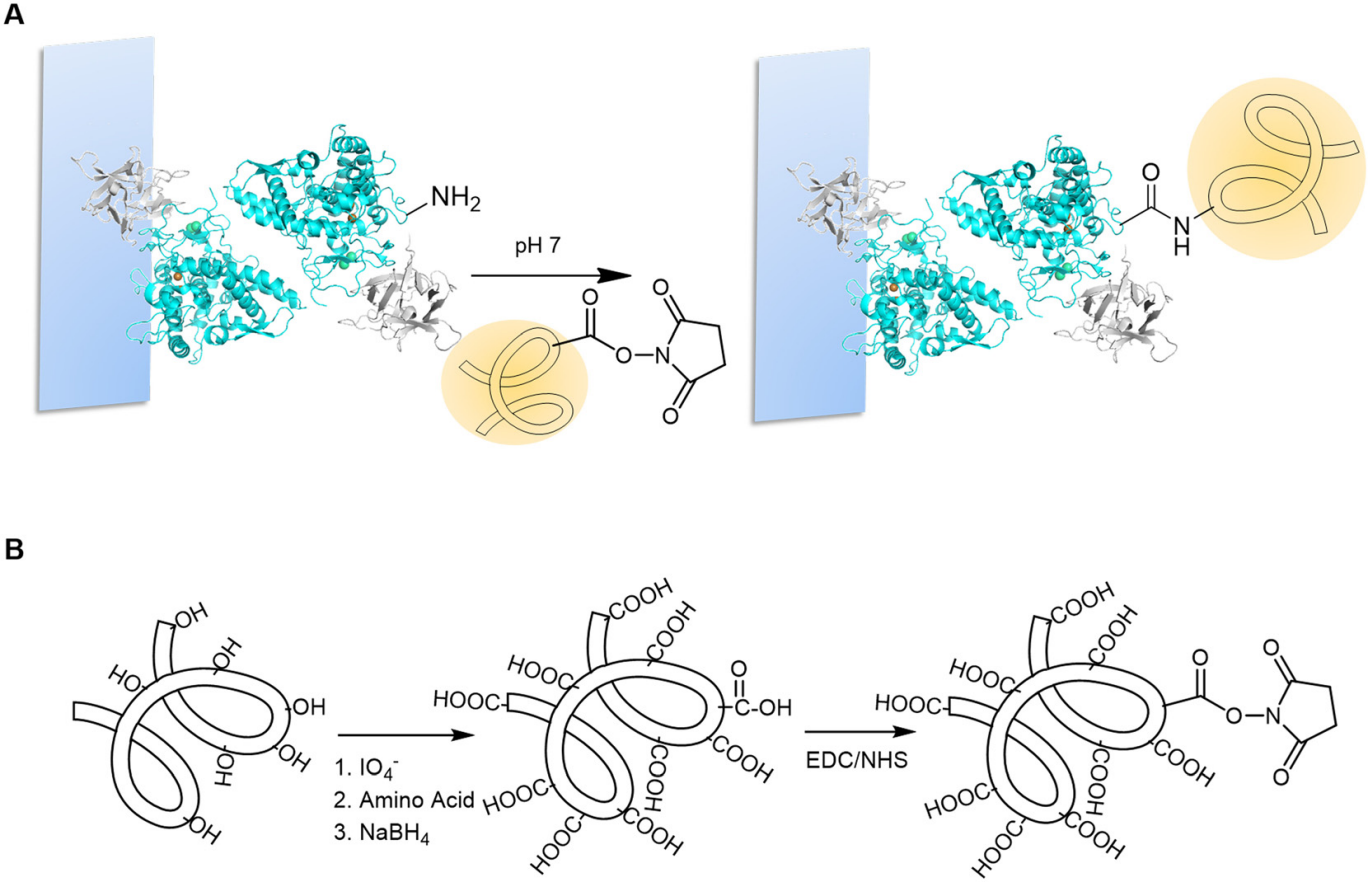
A. Tyrosinase modification on the solid phase. B. Preparation and activation of carboxylated polymers.

The covalent linking was targeted at the free amine groups of tyrosinase. Before the last step of elution (Fig. 3), immobilized AbTyr was used to perform the post-translational modifications. The solid phase makes this process faster and easier, avoiding extra steps to separate the enzyme from free excess polymers (Fig. 4A). This was performed using the EDC/NHS strategy, by which the carboxylic groups of the polymers or peptides were activated by EDC, and then stabilized by *N*-hydroxy succinimide (NHS). This allows them to react with the amine groups of the enzyme, forming a covalent bond. As glycans, tailor-made dextran aldehyde (2000 kDa or 6 kDa) modified with aspartic acid, lysine or glycine (Fig. 4B),^25^ hyaluronic acid, and polygalacturonic acid were used. In the case of peptides, several that contained mainly negatively charged (aspartic acid and glutamic acid residues) and/or hydrophobic amino acids (alanine, glycine and phenylalanine) were used. All tyrosinase conjugates were then tested for their enzymatic activity. Most of them retained a similar diphenolase activity, except for Tyr-Polygal, which lost its activity.

**Fig. 5:**
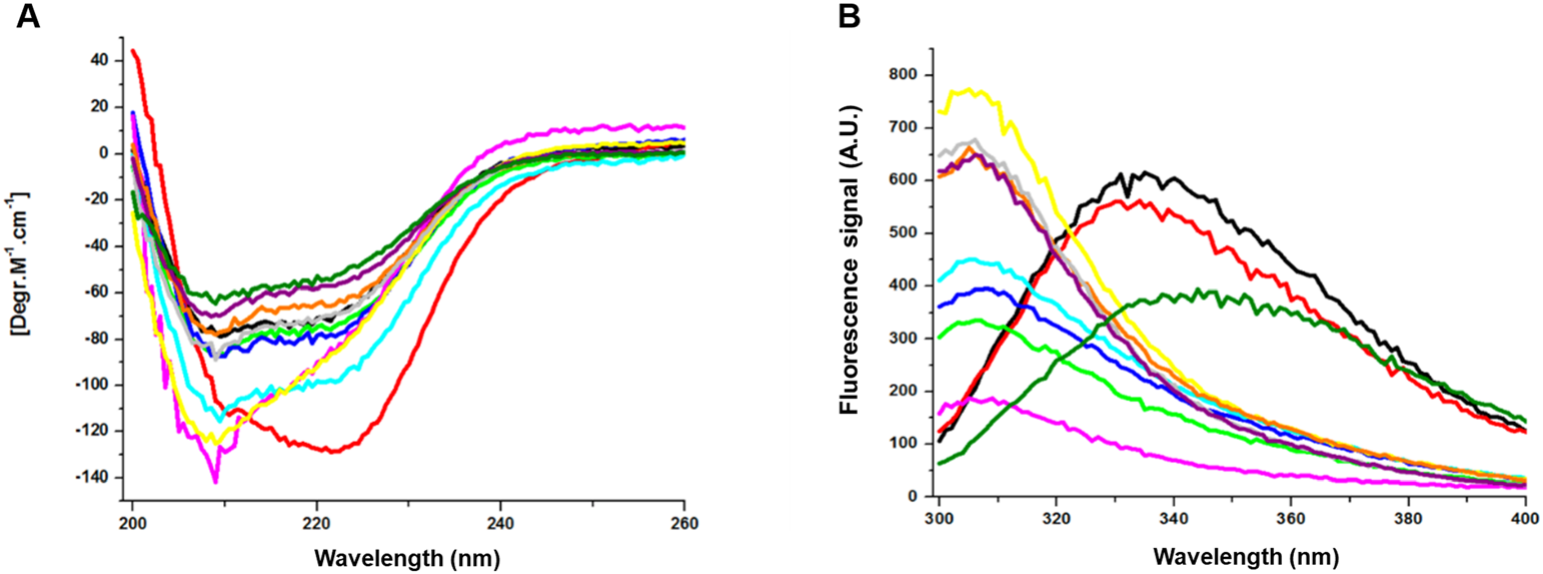
A. Circular dichroism of AbTyr samples. B. Fluorescence spectroscopy of AbTyr samples. NoAct: AbTyr without enzymatic activity after heat deactivation. Unmodified tyrosinase (black), SC18: C18 supernatant (red), Tyr without activity (dark green), Tyr-DextAsp-2000 kDa (pink), Tyr-DextAsp-6 kDa (light green), Tyr-DGED (light blue), Tyr-Hyal (yellow), Tyr-AAGTA (grey), Tyr-FDLG (purple), and Tyr-FFD (orange).

Their stability in different temperatures or the presence of inhibitors or organic solvents was also evaluated. Significant changes were found in the case of Tyr-DextAsp-2000 kDa, whose enzymatic activity significantly decreased at 45 °C compared to AbTyr. When exposed to acetonitrile 40% (v/v), AbTyr lost its activity, while some modifications retained activity: Tyr-DextAsp-6 kDa and Tyr-DGED at 5%; Tyr-Hyal, Tyr-FDLG and Tyr-AAGTA at 15–20%, and Tyr-DD at 40% of its initial enzymatic activity. Far-circular dichroism (CD) was performed to study the effect of modifying the secondary structure, and fluorescence assays were used to analyse the effect on the tertiary structure (Fig. 5).

According to the far CD spectrum (Fig. 5A), the content of α-helix secondary structure in AbTyr decreased after the modification process with FDLG and FFD peptides. The structure was conserved after modification with DextAsp-6 kDa and AAGTA, DD peptides. Conversely, an increase in the content of α-helix secondary structure was observed in the bioconjugate containing the DextAsp-2000 kDa, Hyal or DGED peptide. Additionally, in the fluorescence spectrum, there was a displacement of the maximum of 30 nm (from 335 to 305 nm) for most of the bioconjugates (Fig. 5B). These data suggested that the presence of the polymer also alters the tertiary structure of the enzyme.

### 2.4. Cellular viability

The enzyme and polymers were evaluated in cytotoxicity assays. The results showed that the produced tyrosinase (AbTyr) was found to be non-toxic to cells at up to 2 μM concentrations. The polymers Dex6000 and Dex2M also showed no toxicity up to this concentration, confirming similar results for the bioconjugate. This shows that even the larger polymers were safe (Fig. S1†).

### 2.5. 3CLpro inhibition by AbTyr

**Fig. 6:**
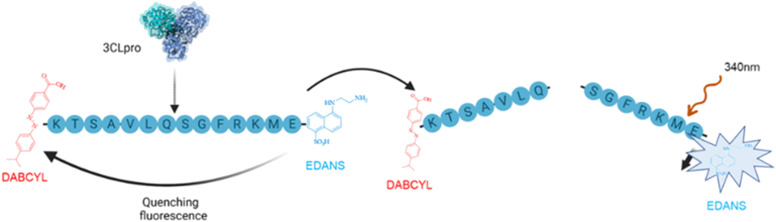
3CLpro *in vitro* FRET fluorescence assay.

Purified AbTyr and their new bioconjugates were tested as an inhibitor of 3CLpro protease activity using FRET experiment assays (Fig. 6), which showed the inhibitory sample concentrations at which 3CLpro protease activity is reduced to 50% (IC_50_) and to 90% (IC_90_).

**Fig. 7:**
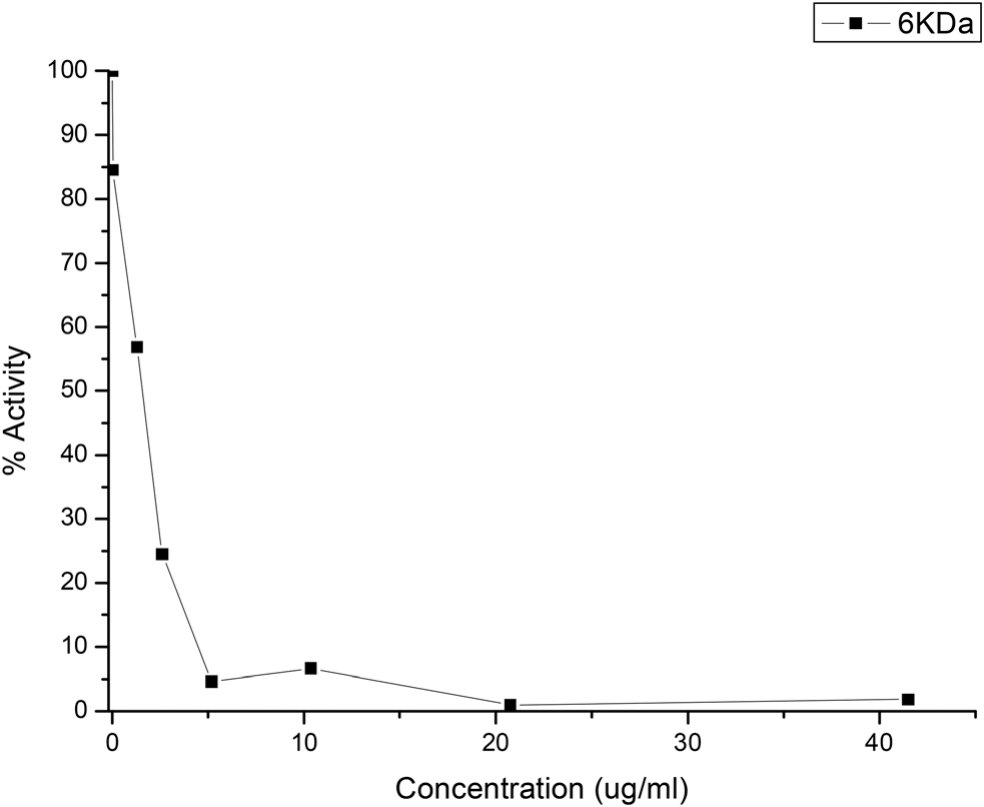
Inhibitory activity of Tyr-DextAsp-6 kDa against the 3CLpro protease activity.

Quercetin was used as the positive control (Table 2, entry 1). AbTyr showed slight inhibition, with IC_50_ > 50 μg mL^−1^ (Table 2). Tyrosinase modifications with cationic polymers did not present significative inhibition of 3CLpro activity, while carboxylated polymers showed much better results. The best result was obtained by Tyr-DextAsp-6KDa, with an IC_50_ value of 2.5 μg mL^−1^ and IC_90_ value of 5 μg mL^−1^ (Fig. 7). Other conjugates also showed good inhibition values, such as Tyr-Polygal or Tyr-Hyal with IC_50_ values of 5 and 10 μg mL^−1^, respectively. In particular, the result of conjugated polygal is interesting because it showed loss of diphenolase activity in the spectrophotometric assay against l-DOPA. This good result as a 3CLpro inhibitor might be explained by its monophenolase activity. In the case of peptide modification, the bioconjugate with a hydrophobic tripeptide, Tyr-FFD, showed an IC_50_ value of 7.5 μg mL^−1^. Conversely, when including alanine in the peptide sequence, the lowest inhibition values were observed (Table 2). Free polymers were also tested for inhibition of 3CLpro activity. The negative results confirmed that this inhibition was caused exclusively by the presence of the protein sample (data not shown).

**Table 2:** Inhibitory concentrations of AbTyr against 3CLpro

Polymer	IC_50_ (μg mL^−1^)	IC_90_ (μg mL^−1^)
Quercetin	6.3	57
AbTyr	>50	—
Tyr-DextAsp-6 kDa	2.5	5
Tyr-DextAsp-2 MDa	—	—
Tyr-DextLys-6 kDa	—	—
Tyr-DextGly-6 kDa	—	—
Tyr-Hyal	10	40
Tyr-Polygal	5	20
Tyr-DD	17.5	>40
Tyr-DGED	35	>40
Tyr-FFD	7.5	15
Tyr-AGAG	40	>40
Tyr-FDLG	—	—
Tyr-AAGTA	—	—

These excellent results with the enzyme demonstrate the great potential applicability of this method. Similar and, in some cases, highest inhibitory efficiencies were obtained from some of the recent synthesized peptidomimetics.^27–29^

### 2.6. Antiviral activity of Tyr-DextAsp-6 kDa against SARS-CoV-2

**Fig. 8:**
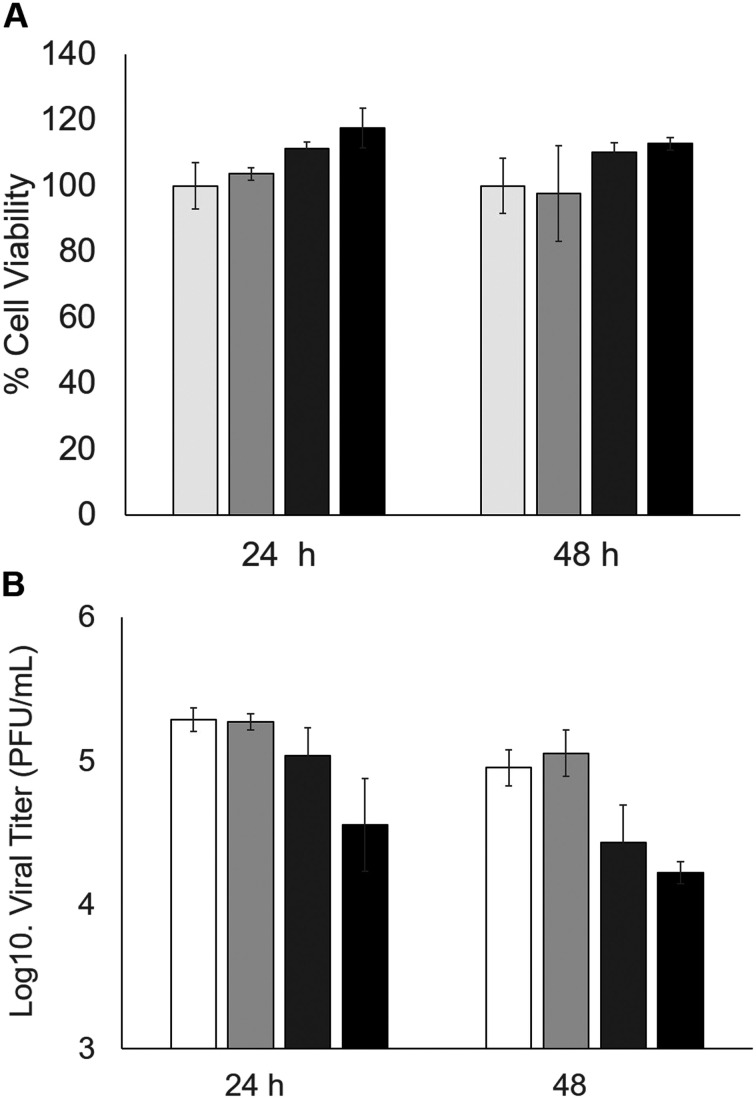
Inhibition of SARS-CoV-2 in Vero cells by Tyr-DextAsp-6 kDa. A) Cell viability. B) Inhibitory effect. Without bioconjugate (white column), 5 μg mL^−1^ (light grey), 50 μg mL^−1^ (dark grey), 100 μg mL^−1^ (black).

Subsequently, the inhibitory capacity of Tyr-DextAsp-6 kDa was evaluated against SARS-CoV-2 in Vero E6 cells (Fig. 8). The impact of varying concentrations of the tyrosinase bioconjugate (5–100 μg mL^−1^) was assessed over an incubation period of 24–48 hours. The initial cell viability assessment demonstrated that the protein bioconjugate did not induce cytotoxicity in any of the tested cells (Fig. 8A). The inhibitory effect of the bioconjugate on SARS-CoV-2 replication was determined by observing a reduction in the viral load relative to that observed in the absence of the bioconjugate. At a lower concentration of 5 μg mL^−1^, the virus was not inhibited even after 48 hours of incubation (Fig. 8B). At a concentration of 50 μg mL^−1^, the virus was effectively inhibited, with a greater reduction observed after 48 hours, resulting in a 0.5 log 10 reduction (66.6% decrease in viral load). This result was obtained at 24 h using a double amount of bioconjugate, resulting in an 88% decrease in viral load after 48 h (Fig. 8B). The results demonstrate that the tyrosinase bioconjugate is effective in inhibiting the SARS-CoV-2 virus.

## 3. Conclusions

In this work, we developed a green and efficient technology to produce enzyme bioconjugates. Starting from a tyrosinase extracted from a natural and cheap source, the product was purified efficiently using a simple chemical modification with tailor-made polymers, all prepared in aqueous media and at room temperature. This enzyme was evaluated as a potential antiviral protein therapeutic *via* its inhibition capacity of the 3CLpro protease *in vitro*. Although this enzyme has recently exhibited promising antiviral activity against hepatitis C virus, its effect against 3CLpro was moderate. The new bioconjugates demonstrated promising inhibitory activity, with IC_50_ values as low as 2.5 μg mL^−1^ for Tyr-DextAsp-6 kDa. They were also effective in inhibiting SARS-CoV-2 replication in infected cells, with an 88% decrease in virus load. These results clearly demonstrate the potential of these bioconjugates as a promising new pharmaceutical agent, particularly in combination with other antiviral drugs. Polymer modification also enhanced the stability and cell penetration capacity of the enzyme, making it an attractive avenue for further investigation. One potential approach is to design a new type of protein bioconjugate combining this modification with interesting peptides or peptidomimetics as novel structures. This could also enhance biocompatibility and cell penetration properties, while significantly improving efficiency against the virus. This technique also offers the potential for expansion and applicability against other viruses.

## 4. Experimental section

### 4.1. Tyrosinase extraction from *Agaricus bisporus*

Mushrooms were purchased from supermarkets Mercadona (Champinter), Hiber (Neofungi) and Simply (Laumont). The protocol for the tyrosinase extraction is as follows: 15 g of mushrooms without the stipes were chopped and put under agitation in 50 ml of cold acetone for 30 min in the Bunsen rod stirrer AGV-8, and then centrifuged at 7000 rpm and 4 °C for 20 min using the centrifuge Biocen 22R. The supernatant was discarded, and the mushrooms were put under agitation in 50 ml of cold water for 1 h in the Nahita DJ-1 rod stirrer. The mix was centrifuged at 8000 rpm and 4 °C for 20 minutes, the pellet was discarded, and 15 g of ammonium sulfate (Sigma-Aldrich) was slowly added to the supernatant under gentle agitation using a magnet stirrer. This protein extract was centrifuged at 8000 rpm and 4 °C for 40 min, and the pellet was stored at −20 °C. The pellet was resuspended in distilled water, and dialysis was performed using a tubing cellulose membrane (D9652, molecular weight cut-off = 14 kDa, Sigma-Aldrich) in 1 L water or sodium phosphate (25 mM) buffer at pH 7 for 30 min, which was repeated 3 times. Protein solutions after dialysis were stored at 4 °C.

### 4.2. Tyrosinase enzymatic activity assay

The enzymatic activity of tyrosinase was measured in the presence of 1 ml of 1 mM l-3,4-dihydroxy-phenylalanine (l-DOPA, Sigma-Aldrich) in sodium phosphate buffer (0.1 M, pH 7) at room temperature. The increase in absorbance at 475 nm after adding 25 μL of sample was measured using the V-730 spectrophotometer (Jasco). An increase of 0.001 of absorbance in 1 minute was defined as an enzymatic activity unit (U). Enzymatic activity was also tested in the presence of 1 mM l-ascorbic acid or hydroquinone (Sigma-Aldrich), or 40% (v/v) acetonitrile.

### 4.3. SDS-PAGE electrophoresis

Sodium dodecyl sulphate-polyacrylamide gel electrophoresis (SDS-PAGE) was performed using the system PerfectBlue™ Double Gel System Twin S from Peqlab, with the PS300B Volt Power Supply from Hoefer at 170 V and constant amperage. A 12% resolving gel (acrylamide 12%, 0.375 M Tris-HCl pH 8.8, 0.1% SDS, 0.05% APS, TEMED) and 5% stacking gel (acrylamide 5%, 0.136 M Tris-HCl pH 6.8, 0.1% SDS, 0.05% APS, TEMED) were used, and the SDS-PAGE was run in a buffer containing 0.2 M glycine, 0.25 M Tris-HCl (pH 8.8), and 1% SDS. The marker used for the gels was unstained protein molecular weight marker from Thermo Scientific. Gels were stained with Coomassie brilliant blue (0.25%), and unstained with a solution of 43% methanol and 7.5% acetic acid. Samples were prepared in a 1 : 1 solution with 2× loading buffer (80 mM Tris-HCl (pH 6.8), 4% SDS, 20% glycerol, 10% 2-mercaptoethanol, bromophenol blue 0.02%). Silver staining was performed following the Protocol for Silver Staining of Gels from Alphalyse, using sodium sulfate instead of sodium thiosulfate.

### 4.4. Protein adsorption on solid supports

Tyrosinase hydrophobic adsorption was tested on different supports: Butyl-Sepharose (C4), Octyl-Sepharose (C8, from GE Healthcare), geranyl-functionalized carboxymethylcellulose (G-CM), Octadecyl Sepabeads (C18, from RESINDION) and Epoxy-C18 (E-C18, from Purolite). The adsorption was performed using 4 ml of sample (0.4 mg ml^−1^ or 0.25 mg ml^−1^) with 0.3 g of solid support, in water or in sodium phosphate (25 mM or 100 mM, pH 7) in a roller for 1 h. Different concentrations of Triton X-100 (0.01–0.25%) were used for the desorption of the proteins from the solid phase after an incubation period of 1 h.

### 4.5. Covalent binding of polymers and peptides

Proteins were modified using the following peptides: Ac-DD, Ac-DEGD, Ac-FFD, Ac-AGAG, Ac-FDLG and Ac-AAGTA from GenScript, and the polymers containing carboxylic groups: commercial polymers: hyaluronic acid (8–15 kDa), polygalacturonic acid (25–30 kDa) and polyethyleneimine 25 kDa from Sigma-Aldrich, and tailor-made synthesized dextran-aspartic 6 kDa, dextran-aspartic 2 MDa, dextran-lysine 6 kDa and dextran-glycine 6 kDa.^18^ The different polymers were incubated in the presence of 10 eq. *N*-ethyl-*N*′-(3-(dimethylaminopropyl)carbodiimide (EDC)) (Tokyo Kasei) and 15 eq. *N*-hydroxysuccinimide (NHS) (Sigma-Aldrich) in water at pH 4.5 for 1 h. 50 or 100 eq. of activated polymers were incubated with 1.6 mg of proteins supported on a solid phase of C18 (0.3 g) overnight in sodium phosphate (50 mM) or water at pH 7, and then washed with sodium phosphate buffer (25 mM, pH 7). Modified proteins were eluted from the support using 0.05% or 0.1% Triton X-100.

### 4.6. 3CLpro inhibition assay

*In vitro* 3CLpro catalytic activity was determined using a fluorescence resonance energy transfer (FRET) assay with the peptide (Dabcyl)-KTSAVLQSGFRKME-(Edans)-NH_2_ (Biosyntan GmbH) as substrate. A concentration of 0.2 μM of protease was used in sodium phosphate (50 mM), NaCl (150 mM, pH 7), and the enzymatic reaction was started by adding the peptide at a concentration of up to 20 μM in a volume of 100 μL. In the case of quercetin (positive control), 2 μM of enzyme and 20 μM of substrate were used. The microplate reader FluoDia T70 (Photon Technology International) was used to continuously measure fluorescence for 60 minutes at an excitation wavelength of 380 nm and emission length of 500 nm. Protease activity was quantified as the increase in fluorescence. Inhibition of this activity was defined as the difference between the protease activity in the presence or absence of the inhibitor, and was evaluated by adding up to 400 μg mL^−1^ of these compounds.

### 4.7. Circular dichroism and fluorescence spectroscopy

Circular dichroism of the tyrosinase samples was measured using the Chirascan spectropolarimeter (Applied Photophysics) at 25 °C. Far-UV spectra were recorded in a 0.1 cm path-length cuvette at wavelengths between 200–260 nm, and a protein concentration of 10 μM in phosphate buffer saline at pH 7.2. Fluorescence spectroscopy measurements of 2 μM of each protein sample were obtained using the Varian Cary Eclipse fluorescence spectrophotometer (Agilent Technologies) with an excitation wavelength of 280 nm, and recording the emission spectra between 300 nm and 400 nm.

### 4.8. Cells and viruses

Vero E6 (VERO C1008 [Vero 76, clone E6, Vero E6], African green monkey kidney epithelial cells, kindly provided by Luis Enjuanes at CNB, Spain) and Calu3 2B4 clone (human bronchial epithelial cells with increased ACE-2 expression, kindly provided by Kent Tseng at UTMB, USA and Luis Enjuanes at CNB, Spain) cell lines were grown at 37 °C in DMEM medium supplemented with 5% or 20% fetal calf serum (FCS). SARS-CoV-2 isolate MAD6 (Wuhan-Hu-1-like strain, kindly provided by Luis Enjuanes at CNB, Spain) was propagated in Calu3 2B4 cells, while its titration was done in Vero E6 cells. To measure SARS-CoV-2 titers by plaque assay, viruses were subjected to serial 10-fold dilutions, added to confluent Vero E6 cell monolayers, and incubated for 60 min at 37 °C. The medium was removed, and cells were overlaid with DMEM containing 0.3% carboxymethyl cellulose (CMC) and 2% FBS. At 72 hpi, cells were fixed with 10% formaldehyde and stained with crystal violet.

### 4.9. Cell viability assays

The compound were diluted in DMEM medium supplemented with 2% FCS. Calu3 2B4 cell monolayers were grown on 96-well plates before the addition of the compound at different concentrations (0, 5, 50, 100 μg mL^−1^). After 24 and 48 h of incubation, cell viability was measured using the Cell Titer Glo kit (PROMEGA).

### 4.10. SARS-CoV-2 infections and antiviral treatment

Calu3 2B4 cell monolayers were grown on multiwell plates 24 h before use. Cell monolayers were infected with SARS-CoV-2 at a multiplicity of infection at 1 plaque forming unit (PFU) per mL for 1 h. The infections were performed at biosafety level-3 (BSL-3). The virus inoculum was removed, and cells were washed twice with medium before the addition of drug-containing fresh medium (supplemented with 2% FCS). Cultures were then incubated for 24 and 48 h. Total virus (intracellular and extracellular) production was evaluated by plaque assay on Vero E6 cells.

## Conflicts of interest

There is no conflict of interest to declare.

## Supplementary Material

MD-015-D4MD00289J-s001

## Data Availability

The data supporting this article have been included as part of the ESI.†
