# Supplementary material for: Inhibition of SARS-CoV-2 3CLpro by chemically modified tyrosinase from Agaricus bisporus
Source: RSC Med Chem. 2024 Sep 16;15(12):4159–67. doi: 10.1039/d4md00289j (PMC11451904; doi:10.1039/d4md00289j)
Supplement: MD-015-D4MD00289J-s001 [file MD-015-D4MD00289J-s001.pdf]

Supplementary Information (SI) for RSC Medicinal Chemistry.
This journal is © The Royal Society of Chemistry 2024

Supplementary information

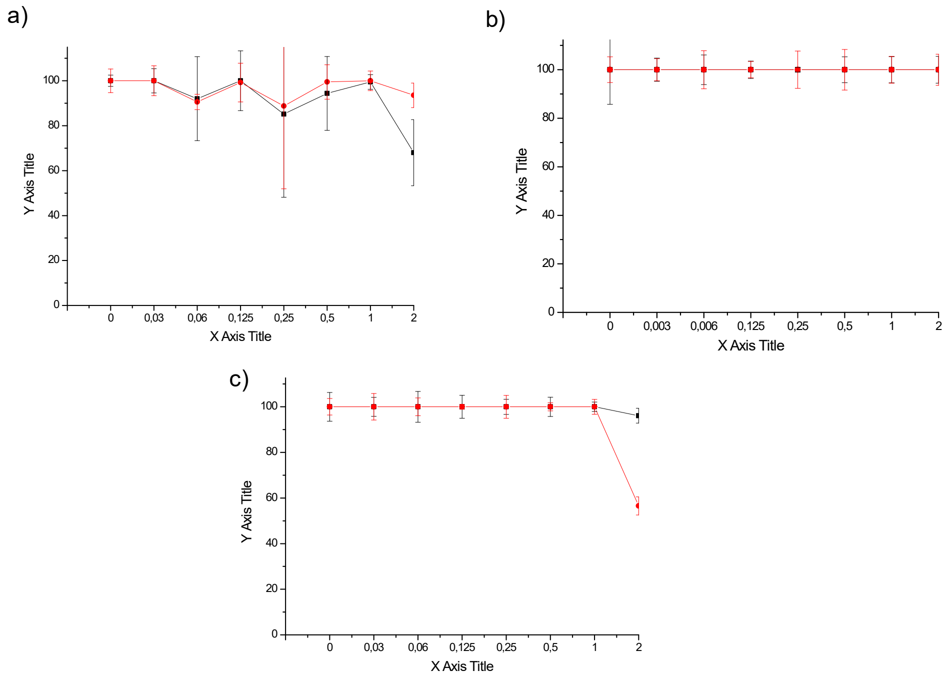

Figure S1. Cytotoxicity of the AbTyr and different polymers against Hub7 cells. A) AbTyr, b) Dex-6000, c) Dext2M. blank (red line), sample (black line).
